# Psychiatric and Psychosocial Outcomes of Bariatric Surgery in Adolescents and Adult Women: A Prospective Cohort Study

**DOI:** 10.3390/jcm15166139

**Published:** 2026-08-07

**Authors:** Marta Herstowska, Ada Przygocka-Pieniążek, Agnieszka Lejk, Jacek Burzyński, Małgorzata Myśliwiec, Łukasz Kaska

**Affiliations:** 1 Department of Pediatrics, Diabetology and Endocrinology, Medical University of Gdansk, 80-210 Gdansk, Poland; 2 Department of Biostatistics and Translational Medicine, Medical University of Lodz, 92-215 Lodz, Poland; jacek.burzynski@umed.lodz.pl; 3 Hospital of the Ministry of the Interior and Administration, 80-104 Gdansk, Poland

**Keywords:** adolescent obesity, bariatric surgery, depression, psychosocial outcomes, mental health

## Abstract

**Background/Objectives**: The rising prevalence of severe adolescent obesity has increased the number of young patients referred for bariatric surgery. The psychiatric and psychosocial profiles of adolescents and of adult women undergoing this treatment remain insufficiently characterized. This study compared preoperative and postoperative psychiatric, psychosocial, and metabolic profiles of adolescents and adult women undergoing bariatric surgery. **Methods**: This prospective cohort study recruited 77 consecutive female patients (32 adolescents aged <18 years; 45 adult women aged 18–25 years) at a single bariatric center in Poland between December 2022 and December 2023. All participants underwent comprehensive preoperative and one-year postoperative assessments including laboratory testing, standardized psychiatric interviews, and psychometric screening with the Beck Depression Inventory (BDI) and the Patient Health Questionnaire-9 (PHQ-9). **Results**: Preoperatively, adolescents were more severely affected for their age (median BMI-for-age z-score 3.53) and waited longer between the first obesity clinic visit and surgery. Adolescents more frequently reported self-harm behaviors and moderate-to-severe depression on the BDI, whereas adult women more often reported psychoactive substance use. Both groups achieved comparable improvements in laboratory and comorbidity outcomes at one year, although relative weight loss was greater in adult women (median total weight loss 37.6% vs. 28.4%, *p* < 0.0001). Postoperatively, a greater proportion of adolescents than adult women improved with respect to social anxiety (50.0% vs. 13.3%, *p* = 0.0007) and BDI depression severity (53.1% vs. 24.4%, *p* = 0.02), whereas a greater proportion of adult women improved with respect to psychoactive substance use (46.7% vs. 12.5%, *p* = 0.003). Generalized anxiety and suicidal ideation declined within both groups, but the extent of improvement did not differ significantly between them. These differences persisted, and for several outcomes became larger, after adjustment for the reduction in BMI, which was smaller in adolescents. **Conclusions**: In this observational cohort, bariatric surgery was associated with distinct psychiatric and psychosocial outcome profiles in adolescents compared with adult women. Because the study was uncontrolled and included no non-surgical comparison group, these findings are hypothesis-generating and cannot establish a causal effect of surgery or of its timing; confirmation in larger, multicenter studies with appropriate comparison groups is required. Comprehensive multidisciplinary perioperative care and tailored postoperative psychological support appear important for both age groups.

## 1. Introduction

Adolescent obesity has reached epidemic proportions, with 2025 data from the WHO and UNICEF estimating that approximately 160 million adolescents worldwide are affected, including roughly 9% of the population in Poland [1,2]. As prevalence continues to rise, conditions once associated primarily with adulthood—type 2 diabetes, hypertension, and dyslipidemia—are now appearing at an earlier age. Childhood obesity also affects mental health, contributing to the development of depressive and anxiety disorders [3]. Obesity with childhood onset moreover persists into adulthood in the majority of affected individuals, compounding both somatic and psychosocial risk across the life course [4].

Given the substantial burden carried by patients with obesity, it is crucial to begin treatment early. Currently, obesity treatment focuses on pharmacological therapy and surgical procedures, as lifestyle-modification strategies alone are associated with only modest and often transient outcomes [5]. Metabolic and bariatric surgery (MBS) results in durable reductions in body mass, along with alleviation of obesity-associated complications. Current guidelines nonetheless position surgery not as a first-line treatment but as an option for patients in whom structured lifestyle modification, dietary intervention, and, where indicated, pharmacotherapy have proved insufficient [6,7,8]. Although MBS is widely used in adults, there remains some reluctance to apply these methods in children.

Outcomes after surgery performed in adolescence have been compared directly with those in adulthood. In the multicenter Teen-LABS study, five years after Roux-en-Y gastric bypass, remission of type 2 diabetes was significantly more frequent among adolescents than among adults, as was remission of hypertension, despite comparable percentage weight change [9]. Ineffectively treated adolescent obesity carries irreversible somatic consequences, as well as lasting psychosocial harm, including depression, self-injurious behaviors, and entrenched body image disturbances [6,7,10]. However, it is estimated that a substantial proportion of eligible patients with obesity are not referred for treatment. This reluctance to offer MBS to younger patients largely reflects apprehension about its possible effects on growth and pubertal development, the threat of nutritional deficiencies and osteoporosis, a heightened risk of alcohol misuse, psychosocial fragility, and uncertainty over whether adolescents can adequately consent to such a decision [7].

The psychiatric and psychosocial profiles of adolescents and of adult women undergoing bariatric surgery nonetheless remain insufficiently characterized. While metabolic benefits of earlier intervention are increasingly documented, adolescence represents a critical developmental window for identity formation, emotional regulation, and social integration—processes that severe obesity profoundly disrupts. How the psychiatric and psychosocial burden of severe obesity differs between adolescents and adult women, in whom it manifests more often through occupational stress, established substance use, and relationship instability, has not been systematically examined. This study compared preoperative and postoperative psychiatric, psychosocial, and metabolic profiles of adolescents and adult women at a single bariatric center in Poland, aiming to describe clinically meaningful differences between these two age groups. The study was not designed to determine the optimal timing of surgery, and no such inference is drawn.

## 2. Methods

### 2.1. Study Design and Participants

This prospective cohort study was reviewed and approved by the Bioethics Committee for Scientific Research at the Medical University of Gdansk (Project Number: NKBBN/868/2021; approval date: 10 December 2021) and was conducted in accordance with the Declaration of Helsinki. We recruited consecutive patients at the Hospital of the Ministry of the Interior and Administration in Gdansk between December 2022 and December 2023. All eligible patients were female; those under 18 years of age at enrollment were classified as adolescents, and those aged 18–25 years as adult women. All participants met accepted eligibility criteria for metabolic and bariatric surgery, namely, a BMI ≥ 35 kg/m^2^ (or ≥120% of the 95th percentile) together with a clinically significant obesity-related comorbidity, or a BMI ≥ 40 kg/m^2^ (or ≥140% of the 95th percentile), whichever was lower, with percentiles evaluated against the Polish national reference [11] and all had previously undergone structured non-surgical treatment—including dietary and lifestyle intervention and, where indicated, pharmacotherapy—that had failed to achieve durable weight reduction [6,7,8,12]. Patients who qualified for bariatric surgery underwent standard preoperative procedures, including specialist consultations with a dietitian, psychologist, psychiatrist, and cardiologist. Inclusion required participation in the psychiatric consultation, at which written informed consent was obtained from all participants (and from legal guardians in the case of minors).

### 2.2. Clinical and Laboratory Assessments

Fasting venous blood samples were obtained preoperatively for analyses including fasting glucose, insulin, C-reactive protein (CRP), glycated hemoglobin (HbA1c), lipid profile, and liver function parameters. BMI was calculated as body weight (kg) divided by height squared (m^2^). Both current and maximal BMI were recorded; maximal BMI was derived from the highest documented body weight. Because BMI is strongly age-dependent during adolescence, absolute BMI is not directly comparable between the two age groups. For participants under 18 years, we expressed BMI relative to the Polish national growth reference derived from the OLA and OLAF studies [11] as the BMI-for-age z-score. Because z-scores compress at the upper extreme of the distribution, the values reported for this severely affected cohort should be read as indicating position well beyond the reference range rather than as finely discriminating between individuals. Absolute BMI is retained for transparency and for comparison with the adult group, but between-group differences in absolute BMI should be interpreted with this limitation in mind.

At the first visit, every participant underwent a complete psychiatric assessment. Diagnoses of psychiatric disorders were established by a board-certified psychiatrist according to DSM-5 criteria. At the first visit and at the one-year follow-up visit, depressive symptom severity was assessed with the Beck Depression Inventory (BDI; 21 items, scored 0–63) and the Patient Health Questionnaire-9 (PHQ-9; 9 items, scored 0–27). Social anxiety was assessed with the Liebowitz Social Anxiety Scale and generalized anxiety with the seven-item Generalized Anxiety Disorder scale (GAD-7); for both instruments, as well as for the BDI and PHQ-9, scores were dichotomized as no/mild or moderate-to-severe. Validated Polish-language versions of all of the aforementioned instruments were used. Psychiatric interviews additionally gathered information on childhood maltreatment, self-harm behaviors, suicidal ideation, and psychoactive substance use. Participants completed questionnaires on demographic background, chronic somatic conditions, sleep quality, and social and cognitive functioning. The one-year postoperative visit was chosen as the primary evaluation point because it approximates the plateau of maximal weight loss after bariatric surgery and matches the assessment window used in previous adolescent bariatric cohorts, permitting direct comparison with those studies [9,13,14].

### 2.3. Statistical Analysis

Nominal variables are reported as counts and percentages; continuous variables as medians with interquartile range (IQR; Q1–Q3). Between-group comparisons of categorical variables used the chi-square test, or Fisher’s exact test where any expected cell count was below five; McNemar’s exact test was applied for paired categorical data. Continuous variables were compared using the Mann–Whitney U test (independent groups) and Wilcoxon signed-rank test (paired comparisons). Effect sizes with 95% confidence intervals (CIs) are reported alongside every comparison. For Mann–Whitney U tests we report the rank-biserial correlation (r_rb) and the Hodges–Lehmann median difference with a bootstrap 95% CI; for Wilcoxon signed-rank tests, the matched-pairs rank-biserial correlation and the Hodges–Lehmann median of paired differences with a bootstrap 95% CI; for independent 2 × 2 comparisons, the odds ratio (OR) with a 95% CI; and for McNemar comparisons, the paired risk difference in percentage points (pp) with a bootstrap 95% CI, which—unlike the discordant-pair OR—remains estimable when improvement occurs in only one direction. All bootstrap intervals use 10,000 resamples with a fixed random seed. BMI-for-age z-scores were derived from the Polish OLA/OLAF 2010 reference for girls, with the published L, M, and S parameters interpolated linearly to exact age [11]. Within-group pre–post comparisons and between-group comparisons address different questions and are reported separately: wherever a difference between adolescents and adult women is claimed, it is supported by a direct between-group test of the proportion of participants who improved (Fisher’s exact test), rather than by contrasting the statistical significance of two within-group tests. To address the possibility that the observed age-group differences merely reflect the differing magnitude of weight loss achieved by the two groups, each between-group comparison was repeated with adjustment for the reduction in BMI between the maximal and the one-year value. Because several outcomes were completely separated—no participant remained in the moderate-to-severe category on the BDI or the PHQ-9 at one year—ordinary maximum-likelihood logistic regression yields infinite estimates, and models were therefore fitted by Firth’s penalized likelihood, which is bias-reduced and remains finite under separation. Confidence intervals are profile penalized-likelihood intervals and *p* values are penalized likelihood-ratio tests. Analyses were conducted in R (version 4.5.2). A *p* value below 0.05 was considered statistically significant. Given the exploratory, hypothesis-generating nature of this study and its limited sample size, no correction for multiple comparisons was applied; consequently, *p* values close to the 0.05 threshold should be regarded as preliminary rather than confirmatory, and the pattern of findings rather than any individual *p* value should guide interpretation.

## 3. Results

### 3.1. Preoperative Characteristics

The analysis included 77 female participants: 32 (42%) adolescents and 45 (58%) adult women. Adolescents exhibited a significantly higher maximal BMI (47.64 [44.99–50.08] vs. 45.01 [39.71–48.83] kg/m^2^, *p* = 0.04; Hodges–Lehmann difference 2.50 kg/m^2^, 95% CI 0.19–5.01) and a higher BMI at the one-year control visit (33.99 [31.06–36.84] vs. 27.55 [24.86–31.46] kg/m^2^, *p* < 0.0001). Relative weight loss at one year was correspondingly smaller in adolescents (total weight loss 28.44% [26.30–31.64] vs. 37.60% [33.63–40.34], *p* < 0.0001). Among the adolescents, the median BMI-for-age z-score was 3.53 [3.40–3.62] at maximal weight, falling to 2.69 [2.35–2.95] at the one-year visit. The interval between the initial obesity clinic visit and the bariatric procedure was nevertheless significantly longer in adolescents (17 [15–20] vs. 9 [8–12] months, *p* < 0.0001).

Physicians less frequently discussed bariatric surgery with younger patients (10 [31.3%] vs. 31 [68.9%], *p* = 0.002; OR 0.21, 95% CI 0.08–0.55) and less often prescribed GLP-1 receptor agonists prior to referral (10 [31.3%] vs. 33 [73.3%], *p* < 0.001; OR 0.17, 95% CI 0.06–0.45). Conversely, adolescents more frequently received structured psychological support during the bariatric treatment process (20 [62.5%] vs. 9 [20.0%], *p* < 0.001; OR 6.67, 95% CI 2.40–18.54), and greater family engagement was observed, including household-wide changes in eating habits.

Adolescents more often had a family history of obesity (32 [100%] vs. 36 [80.0%], *p* = 0.009), particularly on the maternal side (30 [93.8%] vs. 31 [68.9%], *p* = 0.02; OR 6.77, 95% CI 1.42–32.38). Adolescents more frequently reported peer victimization, and adult women psychological violence, although neither difference reached statistical significance (peer victimization 27 [84.4%] vs. 28 [62.2%], *p* = 0.06; psychological violence 9 [28.1%] vs. 23 [51.1%], *p* = 0.07). Preoperative comorbidity was common in both groups and more frequent among adolescents for arterial hypertension (26 [81.3%] vs. 22 [48.9%], *p* = 0.008) and menstrual irregularity (27 [84.4%] vs. 25 [55.6%], *p* = 0.02), with no significant difference for non-alcoholic fatty liver disease (NAFLD; 30 [93.8%] vs. 34 [75.6%], *p* = 0.07) or obstructive sleep apnea (OSA; 22 [68.8%] vs. 24 [53.3%], *p* = 0.26).

**Figure 1:**
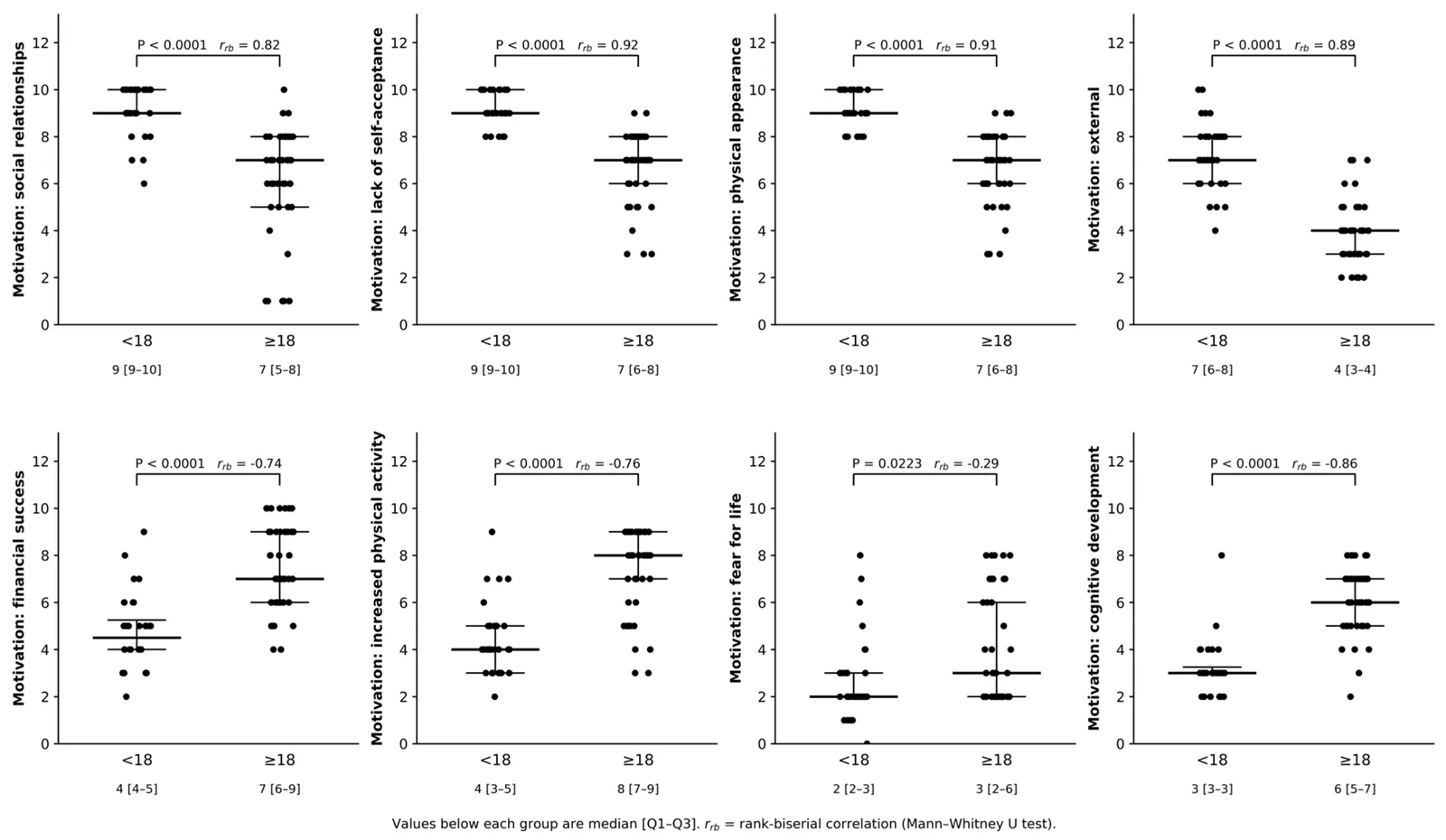
Motivational factors for bariatric surgery by age group. Adolescents more frequently cited lack of body acceptance, physical appearance concerns, and desire to improve social relationships, whereas adult women more often cited physical fitness, general health, and professional advancement.

Motivational profiles differed markedly between the groups (Figure 1); values below are medians [Q1–Q3] on a 0–10 scale, adolescents versus adult women. Adolescents scored higher for lack of body acceptance (9.0 [9.0–10.0] vs. 7.0 [6.0–8.0]), physical appearance (9.0 [9.0–10.0] vs. 7.0 [6.0–8.0]), the wish to improve social relationships (9.0 [9.0–10.0] vs. 7.0 [5.0–8.0]), and the effect of obesity on self-esteem (9.0 [9.0–10.0] vs. 8.0 [7.0–8.0]), and reported greater external pressure from a parent or partner (7.0 [6.0–8.0] vs. 4.0 [3.0–4.0]); rank-biserial correlations ranged from 0.82 to 0.92, all *p* < 0.0001. Adult women scored higher for increased physical activity (4.0 [3.0–5.0] vs. 8.0 [7.0–9.0]), professional or cognitive development (3.0 [3.0–3.2] vs. 6.0 [5.0–7.0]), financial success (4.5 [4.0–5.2] vs. 7.0 [6.0–9.0]), and resolution of comorbidity (3.0 [2.0–4.0] vs. 5.0 [5.0–6.0]); rank-biserial correlations −0.74 to −0.86, all *p* < 0.0001. Fear for life was the only domain in which the groups differed modestly (2.0 [2.0–3.0] vs. 3.0 [2.0–6.0]; r_rb = −0.29, *p* = 0.02). Together with the higher rate of reported peer victimization among adolescents, these patterns indicate that the two groups approach surgery carrying different psychosocial burdens: appearance- and peer-related in adolescents, functional and occupational in adult women.

Detailed group characteristics, environmental factors, and motivational patterns are presented in Table 1 and Figure 1.

Preoperatively, the two groups did not differ in the prevalence of depressive disorder (adolescents: 15 [46.9%] vs. adult women: 20 [44.4%], *p* > 0.99) or in rates of ongoing psychiatric treatment (21 [65.6%] vs. 23 [51.1%], *p* = 0.30). Regarding the anxiety disorder profile, we noted that adolescents more frequently exhibited moderate-to-severe social anxiety (17 [53.1%] vs. 7 [15.6%], *p* = 0.001; OR 6.15, 95% CI 2.12–17.83), whereas adult women showed a higher burden of GAD (moderate-to-severe: 13 [28.9%] vs. 2 [6.3%], *p* = 0.03; OR 0.16, 95% CI 0.03–0.79). Adolescents also reported self-harm behaviors more frequently (18 [56.3%] vs. 14 [31.1%], *p* = 0.049; OR 2.85, 95% CI 1.11–7.30), while adult women more often reported psychoactive substance use (26 [57.8%] vs. 6 [18.8%], *p* = 0.001; OR 0.17, 95% CI 0.06–0.49). On the BDI, moderate-to-severe scores were more common among adolescents (17 [53.1%] vs. 11 [24.4%], *p* = 0.02; OR 3.50, 95% CI 1.33–9.26), although the difference on the PHQ-9 did not reach significance (16 [50.0%] vs. 12 [26.7%], *p* = 0.06). Preoperative characteristics are detailed in Table 2.

### 3.2. Postoperative Outcomes Within Each Group

Both groups demonstrated significant improvements in physical health following surgery, including marked reductions in the rates of arterial hypertension, non-alcoholic fatty liver disease (NAFLD), and obstructive sleep apnea (OSA). Adolescents more often progressed from irregular to regular menstrual cycles than adult women (15 [46.9%] vs. 7 [15.6%], *p* = 0.004; OR 4.79, 95% CI 1.65–13.88). Resolution of hypertension was also more frequent in adolescents (68.8% vs. 42.2%, *p* = 0.04), whereas resolution of NAFLD (84.4% vs. 66.7%, *p* = 0.11) and of OSA (62.5% vs. 40.0%, *p* = 0.07) did not differ significantly between groups. Laboratory parameters improved markedly and to a similar degree in both groups (Supplementary Table S1). In both groups the median aminotransferase activities fell to within the reference range (AST from 71.5 to 36.5 U/L in adolescents and from 63.0 to 32.0 U/L in adult women; ALT from 76.0 to 35.0 and from 68.0 to 28.0 U/L, respectively), as did total cholesterol (329.0 to 187.0 and 327.0 to 198.0 mg/dL) and triglycerides (204.0 to 101.0 and 199.0 to 110.0 mg/dL). Glycated hemoglobin fell from 5.9% to 4.9% in adolescents and from 5.8% to 5.0% in adult women, fasting insulin by two-thirds in both groups, and C-reactive protein by more than half, consistent with resolution of the low-grade inflammatory state associated with severe obesity. HDL cholesterol was the only parameter that did not improve in adolescents (median change 0 mg/dL, *p* = 0.71), whereas a small but significant rise was observed in adult women (+1.5 mg/dL, *p* = 0.006).

Surgery was associated with significant reductions in depressive syndrome in both groups (adolescents: 15 [46.9%] before vs. 3 [9.4%] after, *p* < 0.001, risk difference −37.5 pp, 95% CI −53.1 to −21.9; adult women: 20 [44.4%] vs. 3 [6.7%], *p* < 0.0001, −37.8 pp, 95% CI −53.3 to −24.4) and corresponding decreases in psychiatric treatment rates (adolescents: 21 [65.6%] vs. 3 [9.4%], *p* < 0.0001; adult women: 23 [51.1%] vs. 7 [15.6%], *p* < 0.0001). Both groups showed significant reductions in self-harm behaviors (adolescents: 18 [56.3%] vs. 10 [31.3%], *p* = 0.008; adult women: 14 [31.1%] vs. 5 [11.1%], *p* = 0.004), and patients in both groups reported only mild or no depression on the BDI and PHQ-9 at follow-up.

### 3.3. Between-Group Comparison of Improvement

**Figure 2:**
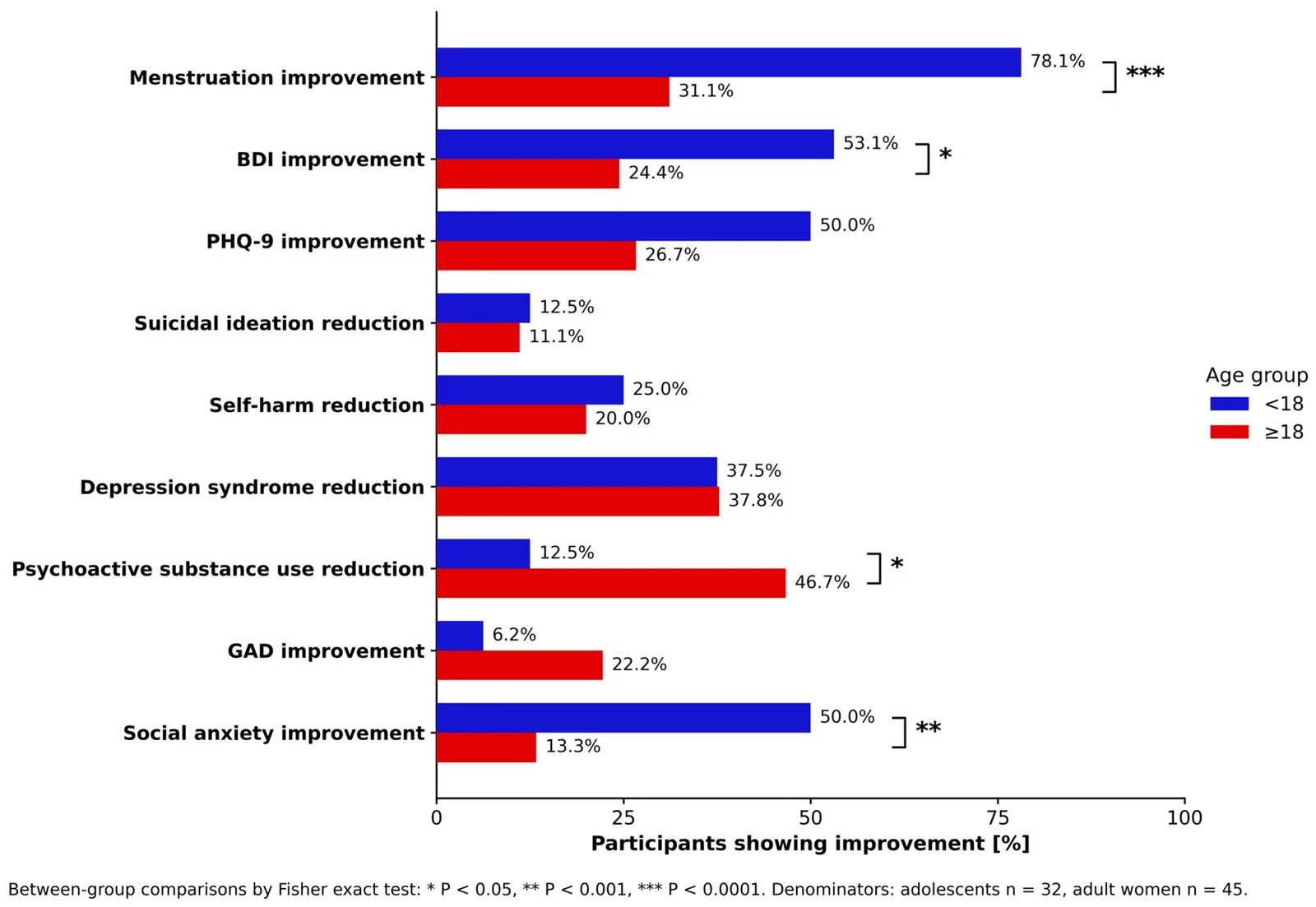
Postoperative improvements in clinical and mental health outcomes by age group. Bars represent the percentage of participants who improved after surgery, stratified by age group (<18 years, n = 32, vs. ≥18 years, n = 45), with exact percentages shown beside each bar. Improvement denotes a change from the affected to the unaffected category between the preoperative and one-year postoperative assessment; for menstrual regularity it denotes a change from irregular to regular cycles. Between-group differences were assessed by Fisher’s exact test and are indicated by brackets (* *p* < 0.05, ** *p* < 0.001, *** *p* < 0.0001).

The postoperative psychiatric trajectory differed between the groups in several respects. Comparing the proportion of participants who improved in each group, adolescents were significantly more likely than adult women to improve with respect to social anxiety (16 [50.0%] vs. 6 [13.3%], *p* < 0.001; OR 6.50, 95% CI 2.15–19.61) and BDI depression severity (17 [53.1%] vs. 11 [24.4%], *p* = 0.02; OR 3.50, 95% CI 1.33–9.26), whereas adult women were significantly more likely to improve with respect to psychoactive substance use (21 [46.7%] vs. 4 [12.5%], *p* = 0.003; OR 0.16, 95% CI 0.05–0.54). For several further outcomes, a within-group reduction was demonstrable in one group but not the other, yet the groups did not differ significantly from each other when compared directly: this applied to generalized anxiety (improvement in 2 [6.3%] adolescents vs. 10 [22.2%] adult women, *p* = 0.11) and to suicidal ideation (4 [12.5%] vs. 5 [11.1%], *p* > 0.99), as well as to self-harm (8 [25.0%] vs. 9 [20.0%], *p* = 0.78), depressive syndrome (12 [37.5%] vs. 17 [37.8%], *p* > 0.99) and PHQ-9 depression severity (16 [50.0%] vs. 12 [26.7%], *p* = 0.05). Adolescents also more frequently reported improvement in social contacts (32 [100%] vs. 37 [82.2%], *p* = 0.02). Within-group pre–post changes are summarized in Table 3, and between-group comparisons of improvement in Figure 2.

### 3.4. Analysis Adjusted for Change in BMI

Because adult women achieved a substantially greater reduction in BMI than adolescents (median 16.54 vs. 13.38 kg/m^2^, *p* < 0.001), each between-group comparison was repeated with adjustment for BMI reduction (Table 4 and Table S2). Adjustment did not attenuate the age-group associations; for most outcomes it strengthened them, because the greater weight loss of adult women had partially masked the differences favoring adolescents. After adjustment, adolescents remained more likely to improve with respect to social anxiety (adjusted odds ratio [aOR] 5.85, 95% CI 1.87–21.32, *p* = 0.002), BDI depression severity (aOR 6.10, 1.95–22.42, *p* = 0.002), and PHQ-9 depression severity (aOR 3.59, 1.24–11.46, *p* = 0.02), while adult women remained more likely to improve with respect to psychoactive substance use (aOR 0.25, 0.07–0.80, *p* = 0.02). Generalized anxiety (aOR 0.37, 0.06–1.65, *p* = 0.19) and suicidal ideation (aOR 1.04, 0.23–4.69, *p* = 0.96) did not differ between the groups either before or after adjustment. It should be noted that every participant with moderate-to-severe symptoms on the BDI or the PHQ-9 preoperatively had improved at one year in both groups, so for these two instruments, the age-group association reflects the higher preoperative symptom burden of adolescents rather than a differential response to surgery.

The same pattern was evident for obesity-related comorbidity. Adolescents were more likely than adult women to experience resolution of arterial hypertension (aOR 4.02, 95% CI 1.43–12.27, *p* = 0.008) and restoration of regular menstrual cycles (aOR 4.64, 1.51–16.28, *p* = 0.007), and after adjustment for BMI reduction, the between-group differences in resolution of non-alcoholic fatty liver disease (aOR 4.85, 1.50–18.07, *p* = 0.008) and of obstructive sleep apnea (aOR 3.92, 1.38–12.18, *p* = 0.010), which had not reached significance in the crude analysis, became statistically significant. Adolescents therefore achieved comparable or better resolution of obesity-related comorbidity despite losing proportionally less weight.

## 4. Discussion

In this prospective cohort study, bariatric surgery in adolescents and adult women was followed by broadly comparable improvements in comorbidity and laboratory parameters but by divergent psychiatric and psychosocial trajectories. Adolescents were more likely than adult women to improve with respect to social anxiety and BDI-rated depressive symptom severity, whereas adult women were more likely to improve with respect to psychoactive substance use. Generalized anxiety and suicidal ideation decreased in both groups without a significant between-group difference. Because this was an uncontrolled observational study without a non-surgical comparison group, these associations cannot be attributed to surgery itself, and less still to its timing; they nonetheless highlight the distinct perioperative needs of each age group [15].

Our data reveal a disparity in how adolescents and adult women reach bariatric surgery. Adolescents waited significantly longer from their first obesity clinic visit to the procedure. Their obesity was also more severe when judged against age-appropriate norms rather than absolute BMI alone, with a median BMI-for-age z-score of 3.53 against the Polish national reference. Physicians less frequently informed adolescents about surgical options and less often prescribed GLP-1 receptor agonists as a preparatory bridge, suggesting that systemic barriers and clinician attitudes—rather than patient characteristics alone—contribute to delayed access to care [12,16]. However, these differences may also be dictated by the later authorization of MBS and GLP-1 analog treatment in the pediatric population. This pattern is consistent with the broader clinical landscape in Poland, where considerable caution persists regarding bariatric treatment in adolescents relative to global trends [16]. Our results may inform a future clinical study, including a larger cohort, to fully characterize the safety profile of those interventions in pediatric population.

Improvements in comorbidity and laboratory parameters were comparably favorable in both groups, consistent with prior reports from the Teen-LABS consortium and with direct comparisons of adolescent and adult surgical cohorts [9,13,17,18]. Relative weight loss was nevertheless smaller in adolescents, so the comparability of these outcomes should not be read as equivalence across every domain. A clinically important concern, however, is that adolescents may show poorer long-term adherence to postoperative recommendations, which could gradually erode initial metabolic gains. The persistence of a higher postoperative BMI in adolescents therefore warrants particular attention during longer-term follow-up. Importantly, however, the smaller weight loss achieved by adolescents does not account for their more favorable psychiatric and comorbidity outcomes: adjustment for the reduction in BMI left every age-group association intact and, for several outcomes, strengthened it, indicating that the differences between the groups are not simply a consequence of differing weight trajectories.

The most clinically significant between-group differences emerged in the psychiatric and psychosocial domains. Both groups showed significant postoperative reductions in depressive syndrome, of closely similar magnitude (risk difference −37.5 pp in adolescents and −37.8 pp in adult women); on the BDI, however, the proportion of participants improving was significantly greater among adolescents. This greater responsiveness may reflect a more plastic cognitive and emotional substrate in younger patients, with fewer entrenched negative self-beliefs, and may also reflect the higher preoperative symptom burden in this group and hence greater scope for measurable improvement [19]. The markedly greater postoperative reduction in social anxiety among adolescents aligns with findings from the Swedish AMOS study [14]. Generalized anxiety, by contrast, improved to a similar extent in the two groups; where it persisted, it may have been sustained by occupational and relational stressors unaffected by changes in body weight [20]. The bidirectional and partly shared biology of obesity and depression may further contribute to the improvements observed in both groups [21]. An important alternative explanation must be considered for two of the preoperative differences we observed. Self-harm and psychoactive substance use follow strong, well-described age gradients in the general population that are independent of obesity: non-suicidal self-injury in females peaks in mid-to-late adolescence and declines thereafter [22], risk factors for adolescent self-harm cluster around peer victimization and family relationships [23], and self-harm presentations in those aged 18–25 years involve alcohol and recreational drugs considerably more often than in those under 18 [24]. The higher preoperative rate of self-harm among our adolescents and the higher rate of substance use among the adult women therefore mirror the age distribution of these behaviors in the wider population, and should not be read as specific to obesity or to bariatric surgery. The same caution applies to other age-patterned variables in this cohort, including relationship status and duration of obesity.

Beyond symptom reduction, surgery was accompanied by age-specific motivational restructuring. In adolescents, gains in self-confidence and reduced fear of rejection were accompanied by the emergence of risk-taking behaviors consistent with normative adolescent development [25], including the possibility of increased impulsive sexual activity [26]. These findings underscore the importance of supplementary cognitive–behavioral support for adolescents specifically aimed at developing adaptive strategies for emotional regulation and impulse control.

Taken together, our findings are compatible with the view that bariatric surgery in adolescents may act not merely as a metabolic intervention but also on the psychosocial consequences of obesity during a critical period of identity formation [27]. This interpretation remains speculative: the present design cannot separate the contribution of surgery from that of the intensive multidisciplinary care, including structured psychological support, that accompanied it, and adolescents in this cohort received such support considerably more often than adult women. Randomized or otherwise controlled studies incorporating non-surgical comparison groups are needed to test it. The integration of GLP-1 receptor agonist therapy as a preoperative bridge may further optimize preparation in this population, although this too requires prospective evaluation [28].

## 5. Limitations

This study has several limitations. First, its single-center design may limit generalizability. Second, the study included only female participants, precluding generalization to male patients. Third, the one-year follow-up may be insufficient for capturing longer-term trajectories, including potential psychiatric symptom recurrence [29,30]. Fourth, some data were collected retrospectively through self-reporting, introducing the possibility of recall bias. Fifth, certain psychiatric diagnoses were established post hoc during the study. Additionally, some psychosocial variables were assessed with non-standardized questionnaires, limiting comparability with other cohorts. Despite these limitations, this study provides a comprehensive preoperative and short-term postoperative characterization of adolescent and young adult female bariatric patients, with longitudinal follow-up at two and five years currently ongoing.

## 6. Conclusions

In this observational cohort, bariatric surgery in adolescents was associated with distinct psychiatric and psychosocial outcomes compared with adult women, particularly in the domains of social anxiety and BDI-rated depressive symptom severity, alongside broadly comparable comorbidity and laboratory outcomes at one year, though with smaller relative weight loss. Because the study was uncontrolled and all participants underwent surgery within a comprehensive multidisciplinary program, these findings cannot establish that surgery caused the observed improvements, nor that performing it earlier is preferable; they should be regarded as hypothesis-generating and require confirmation in larger, multicenter studies incorporating non-surgical comparison groups. Bariatric surgery remains an option for carefully selected adolescents in whom structured non-surgical treatment has proved insufficient, rather than a first-line treatment for obesity. Adolescents require tailored postoperative support addressing impulse control, risk-taking behaviors, and the psychological challenges arising from rapid changes in social identity and self-perception. The integration of GLP-1 receptor agonist therapy as a preoperative bridge warrants prospective evaluation in this population.

## Figures and Tables

**Table 1:** Group characteristics, preoperative comorbidity, healthcare pathway, and socioeconomic factors. Continuous variables are given as median [Q1–Q3] and compared with the Mann–Whitney U test; categorical variables as n (%) and compared with the chi-square or Fisher exact test. HL, Hodges–Lehmann median difference (adolescents minus adult women) with bootstrap 95% CI; OR, odds ratio for adolescents relative to adult women with 95% CI. BMI, body mass index.

Variable		Adolescents (n = 32)	Adult Women (n = 45)	*p*-Value (Effect Size, 95% CI)
Maximal BMI [kg/m^2^]		47.64 [44.99–50.08]	45.01 [39.71–48.83]	0.0433 (HL 2.50, 0.19–5.01)
Maximal BMI-for-age z-score, adolescents only [Polish OLA/OLAF reference]		3.53 [3.40–3.62]	not applicable	not applicable
BMI at one-year control visit [kg/m^2^]		33.99 [31.06–36.84]	27.55 [24.86–31.46]	<0.0001 (HL 5.87, 3.63–7.71)
Change in BMI, control visit vs. maximal [kg/m^2^]		−13.38 [−15.61 to −12.55]	−16.54 [−18.73 to −14.01]	0.0003 (HL 2.68, 1.33–4.04)
Duration of obesity [years]		6 [5–8]	10 [8–13]	<0.0001 (HL −4, −5 to −3)
Total weight loss at one year [%]		28.44 [26.30–31.64]	37.60 [33.63–40.34]	<0.0001 (HL −7.87, −10.36 to −5.74)
Duration of prior obesity treatment [years]		0 [0–1]	2 [1–2]	<0.0001 (HL −1, −2 to −1)
Interval from first obesity clinic visit to surgery [months]		17 [15–20]	9 [8–12]	<0.0001 (HL 7, 6–9)
Information about possible bariatric surgery	Yes	10 (31.25%)	31 (68.89%)	0.0024 (OR 0.21, 0.08–0.55)
	No	22 (68.75%)	14 (31.11%)	
GLP-1 analog prescription prior to surgery	Yes	10 (31.25%)	33 (73.33%)	0.0006 (OR 0.17, 0.06–0.45)
	No	22 (68.75%)	12 (26.67%)	
Psychological support	Yes	20 (62.50%)	9 (20.00%)	0.0004 (OR 6.67, 2.40–18.54)
	No	12 (37.50%)	36 (80.00%)	
Family history of obesity	Yes	32 (100.00%)	36 (80.00%)	0.0086 (OR 16.92, 0.95–302.25)
	No	0 (0.00%)	9 (20.00%)	
Family history of obesity (maternal)	Yes	30 (93.75%)	31 (68.89%)	0.0180 (OR 6.77, 1.42–32.38)
	No	2 (6.25%)	14 (31.11%)	
Family history of obesity (paternal)	Yes	21 (65.62%)	21 (46.67%)	0.1573 (OR 2.18, 0.86–5.56)
	No	11 (34.38%)	24 (53.33%)	
Family higher education attainment	Yes	21 (65.62%)	24 (53.33%)	0.3987 (OR 1.67, 0.66–4.26)
	No	11 (34.38%)	21 (46.67%)	
Family higher education attainment [mother]	Yes	19 (59.38%)	23 (51.11%)	0.6273 (OR 1.40, 0.56–3.49)
	No	13 (40.62%)	22 (48.89%)	
Family higher education attainment [father]	Yes	4 (12.50%)	10 (22.22%)	0.4293 (OR 0.50, 0.14–1.77)
	No	28 (87.50%)	35 (77.78%)	
Victim of physical violence	Yes	5 (15.62%)	13 (28.89%)	0.2792 (OR 0.46, 0.14–1.44)
	No	27 (84.38%)	32 (71.11%)	
Victim of psychological violence	Yes	9 (28.12%)	23 (51.11%)	0.0747 (OR 0.37, 0.14–0.98)
	No	23 (71.88%)	22 (48.89%)	
Peer violence	Yes	27 (84.38%)	28 (62.22%)	0.0622 (OR 3.28, 1.06–10.14)
	No	5 (15.62%)	17 (37.78%)	
Non-alcoholic fatty liver disease (NAFLD)	Yes	30 (93.75%)	34 (75.56%)	0.0732 (OR 4.85, 0.99–23.67)
	No	2 (6.25%)	11 (24.44%)	
Arterial hypertension	Yes	26 (81.25%)	22 (48.89%)	0.0081 (OR 4.53, 1.57–13.11)
	No	6 (18.75%)	23 (51.11%)	
Obstructive sleep apnea (OSA)	Yes	22 (68.75%)	24 (53.33%)	0.2612 (OR 1.93, 0.74–4.98)
	No	10 (31.25%)	21 (46.67%)	
Menstrual cycle irregularity	Yes	27 (84.38%)	25 (55.56%)	0.0157 (OR 4.32, 1.41–13.25)
	No	5 (15.62%)	20 (44.44%)	

**Table 2:** Mental health characteristics prior to surgery. Values are n (%); groups were compared with the chi-square or Fisher exact test. OR, odds ratio for adolescents relative to adult women with 95% CI. BDI, Beck Depression Inventory; BED, binge eating disorder; GAD, generalized anxiety disorder; NES, nocturnal eating syndrome; PHQ-9, Patient Health Questionnaire-9.

Variable		Adolescents (n = 32)	Adult Women (n = 45)	*p*-Value (OR, 95% CI)
Depressive disorder prior to surgery	Yes	15 (46.88%)	20 (44.44%)	>0.9999 (OR 1.10, 0.44–2.74)
	No	17 (53.12%)	25 (55.56%)	
Psychiatric treatment prior to surgery	Yes	21 (65.62%)	23 (51.11%)	0.3008 (OR 1.83, 0.72–4.65)
	No	11 (34.38%)	22 (48.89%)	
GAD	Moderate/severe	2 (6.25%)	13 (28.89%)	0.0293 (OR 0.16, 0.03–0.79)
	No/mild	30 (93.75%)	32 (71.11%)	
Social anxiety	Moderate/severe	17 (53.12%)	7 (15.56%)	0.0011 (OR 6.15, 2.12–17.83)
	No/mild	15 (46.88%)	38 (84.44%)	
Self-harm behaviors	Yes	18 (56.25%)	14 (31.11%)	0.0487 (OR 2.85, 1.11–7.30)
	No	14 (43.75%)	31 (68.89%)	
Psychoactive substance use	Yes	6 (18.75%)	26 (57.78%)	0.0014 (OR 0.17, 0.06–0.49)
	No	26 (81.25%)	19 (42.22%)	
BDI	Moderate/severe	17 (53.12%)	11 (24.44%)	0.0194 (OR 3.50, 1.33–9.26)
	No/mild	15 (46.88%)	34 (75.56%)	
PHQ-9	Moderate/severe	16 (50.00%)	12 (26.67%)	0.0633 (OR 2.75, 1.06–7.16)
	No/mild	16 (50.00%)	33 (73.33%)	
Suicidal ideation	Yes	5 (15.62%)	8 (17.78%)	>0.9999 (OR 0.86, 0.25–2.91)
	No	27 (84.38%)	37 (82.22%)	
NES	Yes	3 (9.38%)	11 (24.44%)	0.1646 (OR 0.32, 0.08–1.26)
	No	29 (90.62%)	34 (75.56%)	
BED	Yes	15 (46.88%)	16 (35.56%)	0.4458 (OR 1.60, 0.63–4.03)
	No	17 (53.12%)	29 (64.44%)	
Bulimia	Yes	3 (9.38%)	6 (13.33%)	0.7277 (OR 0.67, 0.16–2.92)
	No	29 (90.62%)	39 (86.67%)	

**Table 3:** Within-group changes in mental health outcomes from before surgery to the one-year follow-up. Both levels of each outcome are shown; values are n (%) of the group total (adolescents n = 32, adult women n = 45). Each group was compared with itself using McNemar’s exact test. The risk difference (postoperative minus preoperative prevalence of the affected level, in percentage points, pp) is given with a bootstrap 95% CI. These are within-group comparisons only: because a significant change in one group and a non-significant change in the other does not establish a difference between the groups, all between-group comparisons are reported separately in Figure 2. BDI, Beck Depression Inventory; GAD, generalized anxiety disorder; PHQ-9, Patient Health Questionnaire-9.

Outcome		Adolescents (n = 32)			Adult Women (n = 45)		
		Before Surgery	After Surgery	*p* (Risk Difference, 95% CI)	Before Surgery	After Surgery	*p* (Risk Difference, 95% CI)
Depression syndrome	Yes	15 (46.88%)	3 (9.38%)	0.0005 (−37.5 pp, −53.1 to −21.9)	20 (44.44%)	3 (6.67%)	<0.0001 (−37.8 pp, −53.3 to −24.4)
	No	17 (53.12%)	29 (90.62%)		25 (55.56%)	42 (93.33%)	
Psychiatric treatment	Yes	21 (65.62%)	3 (9.38%)	<0.0001 (−56.2 pp, −71.9 to −40.6)	23 (51.11%)	7 (15.56%)	<0.0001 (−35.6 pp, −48.9 to −22.2)
	No	11 (34.38%)	29 (90.62%)		22 (48.89%)	38 (84.44%)	
Psychoactive substance use	Yes	6 (18.75%)	3 (9.38%)	0.3750 (−9.4 pp, −21.9 to +3.1)	26 (57.78%)	5 (11.11%)	<0.0001 (−46.7 pp, −62.2 to −31.1)
	No	26 (81.25%)	29 (90.62%)		19 (42.22%)	40 (88.89%)	
Suicidal ideation	Yes	5 (15.62%)	1 (3.12%)	0.1250 (−12.5 pp, −25.0 to −3.1)	8 (17.78%)	3 (6.67%)	0.0625 (−11.1 pp, −20.0 to −2.2)
	No	27 (84.38%)	31 (96.88%)		37 (82.22%)	42 (93.33%)	
Self-harm	Yes	18 (56.25%)	10 (31.25%)	0.0078 (−25.0 pp, −40.6 to −12.5)	14 (31.11%)	5 (11.11%)	0.0039 (−20.0 pp, −31.1 to −8.9)
	No	14 (43.75%)	22 (68.75%)		31 (68.89%)	40 (88.89%)	
Social anxiety	Moderate/severe	17 (53.12%)	1 (3.12%)	<0.0001 (−50.0 pp, −68.8 to −31.2)	7 (15.56%)	1 (2.22%)	0.0312 (−13.3 pp, −24.4 to −4.4)
	No/mild	15 (46.88%)	31 (96.88%)		38 (84.44%)	44 (97.78%)	
GAD	Moderate/severe	2 (6.25%)	0 (0.00%)	0.5000 (−6.2 pp, −15.6 to 0.0)	13 (28.89%)	3 (6.67%)	0.0020 (−22.2 pp, −35.6 to −11.1)
	No/mild	30 (93.75%)	32 (100.00%)		32 (71.11%)	42 (93.33%)	
BDI	Moderate/severe	17 (53.12%)	0 (0.00%)	<0.0001 (−53.1 pp, −71.9 to −37.5)	11 (24.44%)	0 (0.00%)	0.0010 (−24.4 pp, −37.8 to −13.3)
	No/mild	15 (46.88%)	32 (100.00%)		34 (75.56%)	45 (100.00%)	
PHQ-9	Moderate/severe	16 (50.00%)	0 (0.00%)	<0.0001 (−50.0 pp, −65.6 to −31.2)	12 (26.67%)	0 (0.00%)	0.0005 (−26.7 pp, −40.0 to −13.3)
	No/mild	16 (50.00%)	32 (100.00%)		33 (73.33%)	45 (100.00%)	

**Table 4:** Between-group comparison of postoperative improvement. Improvement denotes a change from the affected to the unaffected category between the preoperative and the one-year assessment; values are n improved/n in group (%). Groups were compared with Fisher’s exact test; odds ratios are for adolescents relative to adult women. Adjusted estimates come from Firth’s penalized logistic regression including the reduction in BMI between the maximal and the one-year value. For the BDI and the PHQ-9, every participant affected preoperatively had improved at one year in both groups, so these estimates reflect the higher preoperative symptom burden of adolescents rather than a differential response to surgery. aOR, adjusted odds ratio; BDI, Beck Depression Inventory; GAD, generalized anxiety disorder; OR, odds ratio; PHQ-9, Patient Health Questionnaire-9.

Outcome	Adolescents (n = 32)	Adult Women (n = 45)	Crude *p* (OR, 95% CI)	BMI-Adjusted *p* (aOR, 95% CI)
Social anxiety (moderate/severe)	16/32 (50.0%)	6/45 (13.3%)	0.0007 (OR 6.50, 2.15–19.61)	0.0020 (aOR 5.85, 1.87–21.32)
BDI depression (moderate/severe)	17/32 (53.1%)	11/45 (24.4%)	0.0157 (OR 3.50, 1.33–9.26)	0.0015 (aOR 6.10, 1.95–22.42)
PHQ-9 depression (moderate/severe)	16/32 (50.0%)	12/45 (26.7%)	0.0540 (OR 2.75, 1.06–7.16)	0.0182 (aOR 3.59, 1.24–11.46)
Psychoactive substance use	4/32 (12.5%)	21/45 (46.7%)	0.0026 (OR 0.16, 0.05–0.54)	0.0187 (aOR 0.25, 0.07–0.80)
Depressive syndrome	12/32 (37.5%)	17/45 (37.8%)	>0.9999 (OR 0.99, 0.39–2.52)	0.9055 (aOR 0.94, 0.34–2.60)
Psychiatric treatment	18/32 (56.2%)	16/45 (35.6%)	0.1031 (OR 2.33, 0.92–5.89)	0.3624 (aOR 1.59, 0.58–4.35)
Self-harm	8/32 (25.0%)	9/45 (20.0%)	0.7811 (OR 1.33, 0.45–3.94)	0.6915 (aOR 1.27, 0.39–4.17)
GAD (moderate/severe)	2/32 (6.2%)	10/45 (22.2%)	0.1081 (OR 0.23, 0.05–1.15)	0.1944 (aOR 0.37, 0.06–1.65)
Suicidal ideation	4/32 (12.5%)	5/45 (11.1%)	>0.9999 (OR 1.14, 0.28–4.64)	0.9561 (aOR 1.04, 0.23–4.69)
Improvement in social relations	32/32 (100%)	37/45 (82.2%)	0.0179 (OR 14.73, 0.82–265.27)	—

## Data Availability

The data are available upon reasonable request.

## Supplementary Materials

The following supporting information can be downloaded at https://www.mdpi.com/article/10.3390/jcm15166139/s1, Table S1: Laboratory and metabolic parameters before and after bariatric surgery; Table S2: Postoperative resolution of obesity-related comorbidity by age group: crude and BMI-change-adjusted comparisons.

## Abbreviations

BDI, Beck Depression Inventory; BED, binge eating disorder; BMI, body mass index; CRP, C-reactive protein; GAD, generalized anxiety disorder; GLP-1, glucagon-like peptide-1; HbA1c, glycated hemoglobin; IQR, interquartile range; NAFLD, non-alcoholic fatty liver disease; NES, nocturnal eating syndrome; OSA, obstructive sleep apnea; PHQ-9, Patient Health Questionnaire-9.
