# Supplementary material for: Psychiatric and Psychosocial Outcomes of Bariatric Surgery in Adolescents and Adult Women: A Prospective Cohort Study
Source: J Clin Med. 2026 Aug 7;15(16):6139. doi: 10.3390/jcm15166139 (PMC13513403; doi:10.3390/jcm15166139)
Supplement: Supplementary file 1 [file jcm-15-06139-s001.zip › jcm-4424232-supplementary.pdf]

Supplementary Table S1. Laboratory and metabolic parameters before and after bariatric surgery.

Parameter	Adolescents (n = 32)			Adult women (n = 45)		
	**Before surgery**	**After surgery**	**p (effect size, 95% CI)**	**Before surgery**	**After surgery**	**p (effect size, 95% CI)**
Clinical and comorbidity outcomes						
Non-alcoholic fatty liver disease (NAFLD), N (%)	30 (93.75%)	3 (9.38%)	< 0.0001 (−84.4 pp, −96.9 to −71.9)	34 (75.56%)	5 (11.11%)	< 0.0001 (−64.4 pp, −80.0 to −48.9)
Arterial hypertension, N (%)	26 (81.25%)	4 (12.50%)	< 0.0001 (−68.8 pp, −84.4 to −53.1)	22 (48.89%)	4 (8.89%)	< 0.0001 (−40.0 pp, −55.6 to −24.4)
Obstructive sleep apnea (OSA), N (%)	22 (68.75%)	2 (6.25%)	< 0.0001 (−62.5 pp, −78.1 to −46.9)	24 (53.33%)	6 (13.33%)	< 0.0001 (−40.0 pp, −55.6 to −26.7)
Menstrual cycle irregularities, N (%)	27 (84.38%)	12 (37.50%)	< 0.0001 (−46.9 pp, −65.6 to −28.1)	25 (55.56%)	18 (40.00%)	0.0156 (−15.6 pp, −26.7 to −6.7)
Laboratory parameters, Me (Q1–Q3)						
AST [U/L]	71.5 (54.75–78.25)	36.5 (22.0–40.25)	< 0.0001 (HL −33.75, −36.50 to −30.50)	63.0 (49.0–78.0)	32.0 (21.0–40.0)	< 0.0001 (HL −32.00, −35.00 to −28.50)
ALT [U/L]	76.0 (58.0–80.0)	35.0 (23.75–37.5)	< 0.0001 (HL −40.00,	68.0 (52.0–88.0)	28.0 (19.0–36.0)	< 0.0001 (HL −40.50,

Parameter	Adolescents (n = 32)			Adult women (n = 45)		
	**Before surgery**	**After surgery**	**p (effect size, 95% CI)**	**Before surgery**	**After surgery**	**p (effect size, 95% CI)**
			−45.00 to −36.00)			−45.00 to −36.00)
Total cholesterol [mg/dL]	329.0 (301.0–373.0)	187.0 (168.0–199.0)	< 0.0001 (HL −148.0, −172.5 to −127.7)	327.0 (260.0–376.0)	198.0 (173.0–216.0)	< 0.0001 (HL −129.5, −149.0 to −107.5)
HDL cholesterol [mg/dL]	43.0 (41.0–45.25)	43.0 (41.0–45.25)	0.7101 (HL 0.00, −1.50 to 1.00)	41.0 (39.0–45.0)	42.0 (40.0–45.0)	0.0058 (HL +1.50, +0.50 to +2.50)
Triglycerides [mg/dL]	204.0 (178.0–230.0)	101.0 (98.0–109.75)	< 0.0001 (HL −101.3, −119.5 to −89.5)	199.0 (184.0–288.0)	110.0 (99.0–132.0)	< 0.0001 (HL −102.5, −117.0 to −89.5)
HbA1c [%]	5.9 (5.50–5.93)	4.9 (4.90–5.12)	< 0.0001 (HL −0.75, −0.90 to −0.65)	5.8 (5.50–5.90)	5.0 (4.90–5.30)	< 0.0001 (HL −0.65, −0.75 to −0.55)
CRP [mg/L]	7.6 (6.33–9.93)	3.3 (3.0–4.3)	< 0.0001 (HL −4.10, −5.55 to −3.10)	7.0 (5.60–9.90)	3.6 (3.0–4.8)	< 0.0001 (HL −3.60, −4.55 to −2.80)

Parameter	Adolescents (n = 32)			Adult women (n = 45)		
	Before surgery	After surgery	p (effect size, 95% CI)	Before surgery	After surgery	p (effect size, 95% CI)
Fasting glucose [mg/dL]	101.0 (96.75–108.0)	94.0 (89.75–97.0)	< 0.0001 (HL −8.00, −9.50 to −6.50)	99.0 (89.0–108.0)	92.0 (89.0–98.0)	< 0.0001 (HL −7.00, −9.00 to −5.00)
Fasting insulin [µIU/mL]	21.0 (18.75–38.25)	7.0 (5.0–14.0)	< 0.0001 (HL −16.00, −20.50 to −14.00)	21.0 (16.0–34.0)	10.0 (6.0–13.0)	< 0.0001 (HL −13.00, −17.01 to −10.50)

Supplementary Table S2. Postoperative resolution of obesity-related comorbidity by age group: crude and BMI-change-adjusted comparisons.

Outcome	Adolescents (n = 32)	Adult women (n = 45)	Crude OR (95% CI)	p	Adjusted OR (95% CI)	Adjusted p
Non-alcoholic fatty liver disease	27/32 (84.4%)	30/45 (66.7%)	2.70 (0.86–8.46)	0.1142	4.85 (1.50–18.07)	0.0078
Arterial hypertension	22/32 (68.8%)	19/45 (42.2%)	3.01 (1.15–7.87)	0.0363	4.02 (1.43–12.27)	0.0079
Obstructive sleep apnea	20/32 (62.5%)	18/45 (40.0%)	2.50 (0.98–6.35)	0.0660	3.92 (1.38–12.18)	0.0097
Restoration of regular menstrual cycles	15/32 (46.9%)	7/45 (15.6%)	4.79 (1.65–13.88)	0.0044	4.64 (1.51–16.28)	0.0069
